# Stroma Targeting Nuclear Imaging and Radiopharmaceuticals

**DOI:** 10.1155/2012/817682

**Published:** 2012-05-21

**Authors:** Dinesh Shetty, Jae-Min Jeong, Hyunsuk Shim

**Affiliations:** ^1^Department of Radiology and Imaging Sciences, Emory University, 1701 Uppergate Drive, C5008, Atlanta, GA 30322, USA; ^2^Winship Cancer Institute, Emory University, Atlanta, GA 30322, USA; ^3^Department of Nuclear Medicine, Seoul National University Hospital, Seoul 110744, Republic of Korea

## Abstract

Malignant transformation of tumor accompanies profound changes in the normal neighboring tissue, called tumor stroma. The tumor stroma provides an environment favoring local tumor growth, invasion, and metastatic spreading. Nuclear imaging (PET/SPECT) measures biochemical and physiologic functions in the human body. In oncology, PET/SPECT is particularly useful for differentiating tumors from postsurgical changes or radiation necrosis, distinguishing benign from malignant lesions, identifying the optimal site for biopsy, staging cancers, and monitoring the response to therapy. Indeed, PET/SPECT is a powerful, proven diagnostic imaging modality that displays information unobtainable through other anatomical imaging, such as CT or MRI. When combined with coregistered CT data, [^18^F]fluorodeoxyglucose ([^18^F]FDG)-PET is particularly useful. However, [^18^F]FDG is not a target-specific PET tracer. This paper will review the tumor microenvironment targeting oncologic imaging such as angiogenesis, invasion, hypoxia, growth, and homing, and also therapeutic radiopharmaceuticals to provide a roadmap for additional applications of tumor imaging and therapy.

## 1. Introduction

**Figure 1:**
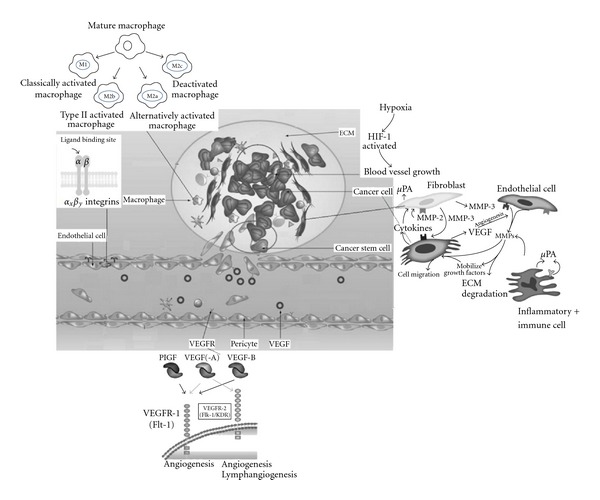
The tumor stroma is critical for tumor growth. Malignant transformation is a multistep process involving profound changes in the normal neighboring tissue, also called tumor stroma. The tumor stroma provides an environment favoring local tumor growth, invasion, and metastatic spreading.

The tumor stroma, consisting of cells, structural proteins, and signaling molecules, which includes fibroblasts/myofibroblasts, glial, epithelial (EC), fat, vascular, smooth muscle, and immune cells along with the extracellular matrix (ECM) and extracellular molecules, is playing a central role in tumor initiation, progression, and metastasis (Figure 1). Growth factor and chemokine production by fibroblasts and immune cells is altered, leading to direct stimulation of tumor cell growth and recruitment of precursor cells, which themselves respond with abnormal growth and proliferation [1]. The unique reciprocal act between the various aspects of the tumor and the microenvironment has been the recent target of molecular strategies for tumor treatment. Targeting the stroma poses several obstacles; however, the level of tumor aggression is greatly influenced by this environment, providing multiple targets for anticancer therapy. The cells associated with stroma are not malignant themselves, which demands successful therapy to aim at phenotypic changes unique to this population, while avoiding normal cells elsewhere. Additionally, malformed tumor vessels contribute to tumor hypoxia, acidosis, and increased interstitial fluid pressures which challenge the delivery of target agents to the stroma. Hence a successful approach requires identification of appropriate targets and efficient delivery methods.

Fibroblasts are the main cellular component of tumor stroma, comprising an integral component of the tumor. Fibroblasts are responsible for the deposition of the fibrillar ECM, which is continually remodeled through a dynamic process of ECM protein production and degradation by fibroblast-derived matrix metalloproteinases (MMPs). Tumor hypoxia influences cytokines and growth factors such as Transforming growth factor-beta (TGF-b), stromal cell- derived factor-1 (SDF-1), matrix metalloproteinase (MMP), vascular endothelial growth factor (VEGF), and hypoxia-inducible factor-1 alpha (HIF-1*α*), which have consistently been shown to directly impact tumor behavior [2–5]. Tumor angiogenesis requires active remodeling of existing cells, which is facilitated by stroma through the expression and secretion of MMPs. Through the secretion of cytokines, chemokines, and other factors, stromal cells are instrumental in creating the unique environment of chronic inflammation and immune tolerance, allowing cancer cells exposure to growth factors.

The potential high sensitivity and specificity of nuclear imaging techniques such as positron emission tomography (PET) and single photon emission computed tomography (SPECT) are an attractive option for medical diagnosis. The high sensitivity of radioisotopes and wide range of biomolecules which are labeled by these radioactive isotopes, such as radioactive halogens (^18^F, ^76^Br, ^77^Br, and ^124^I), [^11^C] and radioactive metals (^111^In, ^99m^Tc, ^68^Ga, and ^64^Cu) made these imaging techniques convenient. In general, radioactive halogens and carbon are widely used to label all kinds of radiopharmaceuticals, but mostly for labeling small molecules, while the radioactive metals are mainly used for labeling large molecules such as proteins, peptides, and antibodies by conjugation of metal chelators. All oncologic imaging tracers are molecularly targeted radiopharmaceuticals based on the tumor biochemistry such as increased metabolism, hyperproliferation, angiogenesis, hypoxia, apoptosis, and specific tumor biomarkers including tumor specific antigens and tumor-specific receptors. This paper will review the tumor microenvironment targeting oncologic imaging and therapeutic radiopharmaceuticals. We hope to provide a roadmap for additional applications of tumor imaging and therapeutic agents which could help researchers and clinicians.

## 2. Targeting Tumor Angiogenesis

### 2.1. Integrin-Targeted Nuclear Imaging

**Figure 2:**
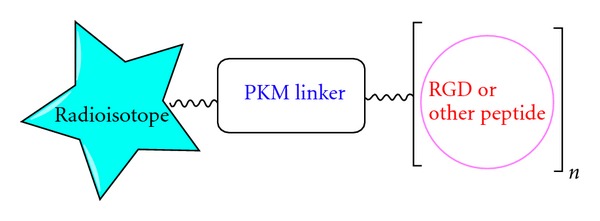
Schematic presentation of the RGD or peptide-conjugated radiotracer design. The pharmacokinetic modifying linker (PKM) is used to improve the radiotracer excretion kinetics. For the metal radioisotopes, bifunctional chelators were attached to targeting molecules, and for [^18^F], molecular synthon is needed to attach biomolecule.

**Figure 3:**
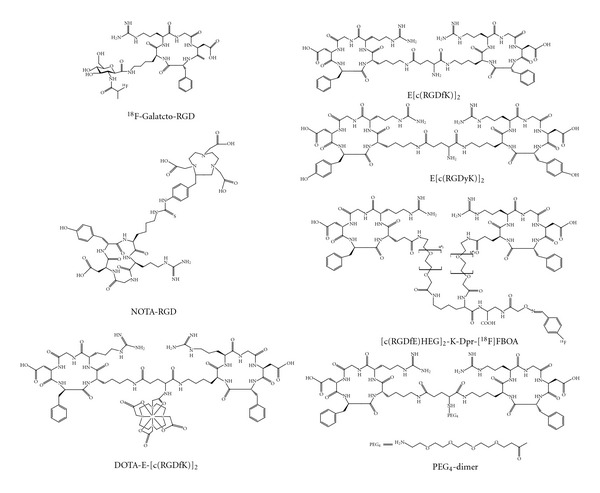
Chemical structures of RGD tracers. ^18^F-labeled galacto-RGD [6], NOTA-RGD [7], and cyclic RGD peptide dimers (E [c(RGDfK)]_2_, E [c(RGDyK)]_2_, [c(RGDfE)HEG]_2_-K-Dpr-[^18^F]FBOA, DOTA-E-[c(RGDfK)]_2_), and PEG_4_-dimer) [8–12].

 Tumor angiogenesis is an essential mechanism for tumor growth and development of metastasis [30, 31]. The angiogenic process depends on vascular endothelial cell migration and invasion and is regulated by cell adhesion receptors. Members of the integrin family play important roles in the regulation of cellular activation, migration, proliferation, survival, and differentiation [32, 33]. Integrins represent a subclass of cell adhesion molecules connecting the cytoskeleton with the ECM or other cells with exposed arginine-glycine-aspartic (RGD) tripeptide sequence [34–39]. Integrins consist of two genetically nonrelated subunits, *α* and *β*, which are noncovalently associated with each other [40–42]. Among all, integrin *α*_v_ *β*_3_ shows overexpression during tumor angiogenesis [30, 43, 44]. Integrin *α*_v_ *β*_3_ is highly expressed on activated endothelial cells but not quiescent endothelial cells of established vessels [45], making it a suitable target for antiangiogenic cancer management. Blocking of these interactions with antagonists leads to detachment of endothelial cells, which drives apoptosis [35]. There have been numerous imaging techniques and therapies based on integrin *α*_v_ *β*_3_ antagonism, including antibodies, peptides, small molecules, and small interfering RNA (siRNA) [46]. Since the introduction of the first *α*_v_ *β*_3_ selective RGD peptides in the 1990s [47, 48], it has been a lead structure for tracer development [8, 49–55]. In general design of RGD peptide-based radiotracers, cyclic RGD peptide serves as the targeting biomolecule to carry radionuclide to the *α*_v_ *β*_3_ integrin site (Figure 2). Various radiolabeled RGD derivatives have been developed for targeting *α*_v_ *β*_3_ integrin expressed during angiogenesis (Figure 3). The pharmacokinetic modifying linker (PKM) is used to improve the radiotracer excretion kinetics. An organic synthon is often used for the F-18 labeling, whereas a multidentate bifunctional chelator (BFC) is used to attach the metallic radionuclides. For an integrin *α*_v_ *β*_3_ targeted radiotracer to be successful, it should have high tumor-specific uptake and tumor-to-blood ratios. It should also be able to distinguish between benign and malignant tumors, to follow tumor growth and metastasis, and to predict therapeutic efficacy in integrin *α*_v_ *β*_3_ positive cancer patients.

The iodinated derivatives of RGD peptide showed receptor-specific tumor accumulation, along with high activity in the liver due to predominant hepatobiliary excretion [50]. The sugar amino conjugate, a galacto-RGD labeled with ^18^F, showed improved pharmacokinetics with significant reduction in the liver accumulation [6]. For further improvement of pharmacokinetics, cyclic RGD was conjugated with tetrapeptides containing hydrophilic D-amino acids [49]. The compounds from this fine tuning approach, specifically ^18^F-Asp3-RGD, showed comparable tumor uptake with ^18^F-galacto-RGD. This approach brought an alternative for PET imaging of *α*_v_ *β*_3_ expression. Another ^18^F-labeled compound, cyclo(-Arg-Gly-Asp-D-Phe-MeVal-) which was synthesized by direct electrophilic fluorination, shows selective integrin-binding affinity. Even though this tracer showed receptor-dependent *in vivo* tumor accumulation, liver and intestine accumulation is similar to the first generation iodinated compounds. Among the radiotracers evaluated in preclinical tumor-bearing models, ^18^F-galacto-RGD [56–59] and ^18^F-AH111585 [60, 61], the core sequence of which was originally discovered from a phage display library (as ACDRGDCFCG), are currently under clinical investigation for visualization of integrin *α*_v_ *β*_3_ expression in cancer patients.

**Figure 4:**
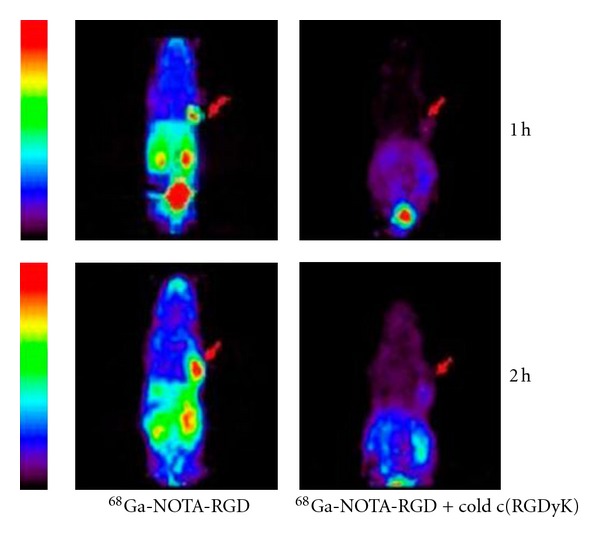
Coronal microPET images of ^68^Ga-NOTA-RGD in mice bearing SNU-C4 xenografts with and without cold c(RGDyK) (60 mg). Arrows indicate tumor positions. Acquisition time was 20 min [7].

There have also been several efforts to develop radiometalated analogues of RGD peptides. These peptides were conjugated with metal chelator at lysine moiety of peptide and the resulting compounds were labeled with ^99m^Tc, ^188^Re, ^90^Y, and recently with ^68^Ga [7, 54, 62]. The gamma scintillation images of ^99m^Tc labeled compound showed clearly contrasting tumor with high tracer uptake in the kidneys. Van Hagen et al. labeled a DTPA-conjugated cyclo(-Arg-Gly-Asp-D-Tyr-Lys-) with ^111^In [55]. Autoradiography and immunohistochemical results demonstrated receptor-specific binding in newly formed vessels. ^68^Ga-labeled derivative of cyclic RGD showed significant specific uptake in tumor xenografted mice (Figure 4). To confirm the specific binding of developed tracer, a blocking study was conducted by injecting cold cyclic RGD before injecting labeled compound and which demonstrated clear inhibition of labeled tracer uptake by tumor cells.

To improve the binding affinity, multimeric RGD peptides have been developed. First, cyclic RGD dimers, such as E [c(RGDfK)]_2_ were developed as diagnostic (^99m^Tc) and therapeutic (^90^Y and ^64^Cu) radiotracers [8, 63, 64]. A linear decapeptide containing two RGD sites labeled with ^99m^Tc has been used for human imaging studies [9]. In imaging studies of melanoma, metastatic lesions exhibited high tumor uptake along with high lung and abdomen tracer uptake. Recently, the ^64^Cu- and ^18^F-labeled E [c(RGDyK)]_2_ were reported as PET radiotracers [10, 65]. Comparison studies found that the RGDfE dimer [c(RGDfE)HEG]_2_-K-Dpr-[^18^F]FBOA had much better targeting capability to its monomeric analogue c(RGDfE)HEG-Dpr-[^18^F]FBOA [11]. Also, many researchers tried to evaluate cyclic RGD tetramers and octamers for angiogenesis imaging. Most of the results revealed the enhancement in the binding affinity and internalization due to peptide multiplicity compared to monomeric or dimeric analogues [11, 66, 67]. Chen et al. studied ^64^Cu and ^18^F labeled cyclic RGD tetramer and octamer for PET tumor imaging [68, 69]. Both *in vitro* assays and *ex vivo* studies showed much higher radiotracer uptake in the case of radiolabeled RGD multimers than their dimeric analogues. Also, as the peptide multiplicity increases, the uptake of radiolabeled multimeric RGD peptides in other organs also significantly increased. They also reported the RGD dimers and tetramers for SPECT imaging of tumor angiogenesis [70, 71]. Even though initial observation credited high binding affinity to peptide multiplicity, there is need to evaluate the impact of multivalency on binding.

A quantum-dot- (QD-) based probe was reported recently for both near-infrared fluorescence (NIRF) and PET imaging of integrin *α*_v_ *β*_3_ expression [72]. In these studies, cyclic RGD peptides and metal chelators were conjugated to a QD for imaging of tumors in living mouse after ^64^Cu labeling. The combination of PET and NIRF imaging overcomes the tissue penetration limitation of NIRF imaging, allowing for quantitative *in vivo* targeted imaging in deep tissue. There are also reports on targeting of integrin *α*_v_ *β*_3_ positive tumor in mice with ^64^Cu-labeled single walled carbon nanotubes (SWNTs) coated with polyethylene glycol (PEG) chains linked to cyclic RGD peptides [73]. In these studies, the intrinsic Raman signatures of SWNTs were used to directly probe the presence of SWNTs in mouse tissues and to confirm the radionuclide-based imaging results. Unlike traditional conjugation methods, in another recent report, ferritin nanocages were loaded with the RGD peptides Cy5.5 and ^64^Cu ferritin for integrin *α*_v_ *β*_3_ targeted PET/NIRF imaging [74]. Recently, engineered knottin peptides, which bind to various integrins with high affinity, were evaluated as molecular imaging agents [75, 76]. For PET and fluorescence imaging in a U87MG human glioma model, Cy5.5 or metal chelator was conjugated to N-terminal of peptide. Results showed that high affinity knottin peptides had higher tumor uptake than the low affinity knottin peptides.

 For dual-modality imaging with SPECT and fluorescence, RGD peptide was labeled with ^111^In and IRDye800 (LI-COR Biosciences) and evaluated in integrin *α*_v_ *β*_3_ positive M21 melanoma xenografts [77]. This study demonstrated the direct comparison of optical and radionuclide imaging for subcutaneous and orthotopic tumors. In a follow-up study, the same probe was used for noninvasive detection of *α*_v_ *β*_3_-positive tumors in mice [78]. The advantage of these techniques over other reported methods lies in better optical imaging resolution and more sensitive detection of the subcutaneous lesions, while gamma scintigraphy allowed for more sensitive detection of deeper structures. Accurate localization of PET probe uptake can be very difficult in some cases due to the absence of identifiable anatomical structures even with PET/CT [79, 80]. A combination of PET/MR can have the answer for many such drawbacks. Highly accurate image registration by MRI can aid in PET image reconstruction. Also, PET/MRI has greatly reduced radiation exposure compared with PET/CT. As a first example to prove these possibilities, poly(aspartic acid)-coated iron oxide (IO) nanoparticles (PASP-IO) coupled to cyclic RGD peptides and metal chelator were reported for integrin *α*_v_ *β*_3_ targeting imaging after labeling with ^64^Cu [81]. PET imaging showed the highest receptor-specific *in vivo* tumor accumulation of ^64^Cu-RGD-PASP-IO after 4 h after injection, while the nontargeted particle showed lower tumor uptake. There are also recent reports on the effort to develop SPECT/CT dual modeling imaging of tumor angiogenesis by ^99m^Tc-labeled nanoparticles [82]. It was found that the tumor-to-muscle signal ratio, after injection of the nanoparticle, was dose dependent and target specific. It was quoted that these nanoparticles can afford highly sensitive and specific localization of tumor angiogenesis, which can be further characterized with high resolution MR neovascular mapping to predict the responsiveness to antiangiogenic therapies.

VEGF is considered a major angiogenic factor responsible for the development of the tumor vasculature network. VEGF-A is the best-characterized member of the VEGF family and is thought to be the most critical regulator of the development of the vascular system in various tumors [83]. Overexpression of VEGF is found in many types of human tumor, which makes VEGF an attractive target for antiangiogenic therapy and blocking the signaling of VEGF in human tumors [84–87]. There have been many reports on different antibodies or chemical molecules binding to VEGF and its receptors [88].

Currently, the most widely used drug in clinic is bevacizumab (Genentech), a humanized monoclonal antibody, which binds to all VEGF isoforms and thereby blocks the VEGF-induced endothelial cell proliferation, permeability, survival, and growth [89, 90]. This drug is approved for clinical use in metastatic colon carcinoma and non-small-cell lung cancer [91, 92]. Despite the promising results of bevacizumab-based therapy, there is a need to monitor *in vivo* VEGF downregulation for the selection of the right patients for bevacizumab-based treatment. To extract these possibilities, two human anti-VEGF antibodies, VG76e (an IgG1 mouse monoclonal anti-VEGF antibody) and HumMV833 (a humanized monoclonal IgG4k antibody), were tested for noninvasive VEGF imaging [93, 94]. ^125^I- and ^124^I-labeled VG76e, which recognizes the 121, 165, and 189 isoforms of human VEGF-A, showed specific tumor targeting in a human fibrosarcoma xenograft model. Maximum uptake was seen after 24 h and declined at 48 h after injection. PET studies in various solid tumors using ^124^I-HuMV833, which binds to VEGF121 and VEGF165, showed variable tumor uptake within patients [94]. These differences are possibly due to the variations in available targets for the antibody between tumor types. There are also reports on bevacizumab labeling with the long-lived PET isotope ^89^Zr and the single *γ*-emitting isotope ^111^In [95–97].

In contrast to the result observed for labeled antibody VG76e, ^89^Zr-bevacizumab has not shown tumor clearance up to 168 h after injection, which indicates the slow clearance of bevacizumab, thus inappropriate as a diagnostic imaging probe. In an alternative approach, Cai et al. reported VEGF-receptor imaging in a human glioma bearing mouse model with ^64^Cu-DOTA-VEGF121 [98]. *In vivo* VEGF receptor imaging could facilitate the evaluation of VEGF-receptor expression, whereas radiolabeled bevacizumab could be used to evaluate VEGF levels. In the subsequent study, researchers showed the feasibility of ^89^Zr-bevacizumab for microPET and CT imaging with enabled quantitative measurement of the tracer in the tumor [99]. There was an interesting report on imaging of liver metastasis in colorectal cancer patients with ^111^In-labeled bevacizumab [100]. These studies revealed an enhanced uptake in the liver metastases in 9 of the 12 patients. But the level of antibody accumulation in tumor lesions varied considerably. There was no correlation found between the level of tracer accumulation and the level of VEGF-A expression in the tissue. Recently there has also been a report on ^64^Cu-labeled bevacizumab for PET imaging of VEGF expression in colorectal cancer xenografts, which showed significant correlation of tumor accumulation of ^64^Cu-DOTA-bevacizumab with VEGF expression as measured by western blot analysis [101]. This study showed higher tumor uptake than the previous report by Nagengast et al., which allowed serial scans due to the longer half-life of the isotope [99]. Even though there are no conclusions on VEGF imaging or therapy, all these exciting reports warrant efforts to learn about new and novel tracers for anti-VEGF therapy.

### 2.2. Integrin-Targeted Radiotherapy

**Figure 5:**
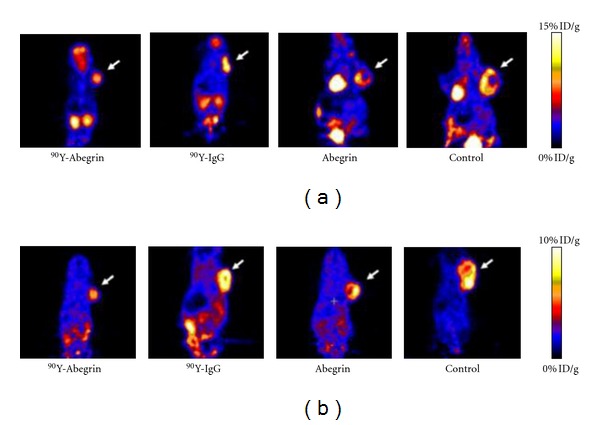
Coronal microPET images and radioactivity accumulation quantification of nude mice bearing U87MG tumors (treated with [^90^Y]-Abegrin, [^90^Y]-IgG, Abegrin, or saline) after i.v. injection of [^18^F]FDG (a) and [^18^F]fluoro-L-thymidine (b).

Integrin *α*_v_ *β*_3_ targeted radionuclide therapy of tumors using monoclonal antibodies (mAbs) and RGD peptides was also investigated by different research groups. Delivery vehicles such as antibodies, RGD peptides, and other small molecules have been investigated for integrin-targeted delivery of cytotoxic drugs and gene inhibitors [46]. The strategy to specifically target multiple sites of disease through radioimmunotherapy minimizes the normal tissue toxicity, which causes cell death of adjacent tumor cells. Researchers tried to eliminate the inevitable production of human anti-murine immunoglobulin antibodies (HAMA) after one to three treatments in patients by utilizing chimeric mAbs or complete humanization of the protein [102]. Though there are several other factors limiting treatment, including slow blood clearance, high uptake in normal organs, and insufficient tumor penetration, there are few studies using antibody for integrin-targeted therapy in progress. To overcome these problems, there has been a new strategy in development of smaller constructs, such as antibody fragments and subfragments, which are capable of binding to the tumor while clearing from normal tissues rapidly [102]. Recently, there was a promising report on therapeutic potential of ^90^Y-labeled humanized anti-integrin *α*_v_ *β*_3_ monoclonal antibody (^90^Y-Abegrin), which was evaluated in U87MG glioma xenograft models [103]. Imaging studies revealed a reduction of cell proliferation, metabolic activity, and DNA synthesis in the ^90^Y-Abegrin-targeted group (Figure 5).

 Low-molecular-weight peptides show fast blood clearance and rapid tumor penetration in contrast to monoclonal antibodies. Even though in recent years structurally modified RGD peptides and analogues were used as the integrin *α*_v_ *β*_3_-targeting vehicles, there are very few reports on therapeutic tumor targeting. One such report on radiolabeled dimeric RGD peptide E [c(RGDfK)]_2_ showed significant increased survival in the case of ^90^Y-DOTA-E [c(RGDfK)]_2_-injected mice compared to untreated mice in an ovarian cancer mouse model [8], but follow-up study failed to attain therapeutic efficacy by increasing the number of injections [104]. There are also reports on ^90^Y-labeled tetrameric RGD peptides for integrin *α*_v_ *β*_3_-targeted internal radiotherapy in mouse tumor xenografts. Also, one more report suggests that the pharmacokinetically improved RGD dimers with PEG4 and Gly3 linkers labeled with ^90^Y showed significant antitumor vasculature effects for integrin *α*_v_ *β*_3_-positive tumors [105]. In another report, the tumor therapeutic potential of ^90^Y/^111^In-labeled monomeric RGD peptide was evaluated in a human ovarian cancer xenograft, and it was claimed that the RGD monomer can be used for fractionated therapy without major toxicity. But due to the lower tumor uptake of the RGD monomer, multiple-dose administration was necessary to achieve therapeutic efficacy [106]. Recently, ^177^Lu labeled two knottin peptides (2.5D and 2.5F), and RGD peptides targeting a range of integrins (*α*_v_ *β*_3_/*α*_v_ *β*_5_/*α*_5_ *β*) were tested for potential radiotherapy in a mouse model of human glioma [107, 108]. ^177^Lu-DOTA-2.5F showed much better *in vivo* results in integrin-positive tumors as a radionuclide therapeutic agent.

 Integrin-targeted radiotherapy by nonpeptide antagonists has also been reported. DOTA-conjugated nonpeptide integrin *α*_v_ *β*_3_ antagonist (TA138) was labeled with ^90^Y and ^177^Lu and tested in an adenocarcinoma model [109]. Biodistribution studies showed similar tumor uptake for both ^111^In-TA138 and ^90^Y-TA138. Results demonstrated a slowing of tumor growth and a regression of tumors.

Theranostics for cancer therapy using radiolabeled peptide derivatives has been attempted recently [110, 111]. For example, octreotide derivatives were used for imaging neuroendocrine tumor patients after labeling with ^68^Ga or ^111^In, and then were administered for therapy after labeling with ^90^Y or ^177^Lu clinically [112–115]. However, clinical results of RGD or knottin labeled with therapeutic radionuclides have not been reported yet.

## 3. Targeting Tumor Invasion

### 3.1. MMP

 The matrix metalloproteinase family shares specific functional and structural components necessary for extracellular secretion and activation of the enzyme. MMP family members are classified on the basis of additional protein domains such as hemopexin or a fibronectin-like region that contribute to their individual characteristics [116]. During active tissue remodeling, MMPs are rapidly transcribed, secreted, and activated [117]. Tumor-associated MMP expression and activity includes a major contribution of surrounding stromal cells. In epithelial cancers, most of the upregulated MMPs are expressed by the host stromal cells [118]. MMPs can also be expressed by tumor cells. MMP7 is commonly expressed in adenocarcinomas, and several MMPs are expressed in the malignant epithelium of tumors that have undergone an epithelial-to-mesenchymal transformation [119]. MMP expression is upregulated in malignant cancers compared to normal, benign, or premalignant tissues [120–122]. MMPs are often associated with the removal of the ECM barrier to allow cancer cells to invade and metastasize [123]. These enzymes are secreted and activated in the extracellular environment, avoiding the need to transfer the probe to intracellular compartments, which is a great advantage when considering MMP as a molecular target. Compared to the probes binding to targets in a 1 : 1 fashion, there is an advantage of signal amplification due to MMP's catalytic activity at physiological pH. Even though MMPs were considered attractive targets for the development of anticancer drugs, much attention was directed to the design and synthesis of MMP inhibitors (MMPis) with the first clinical result reported in the 1990s [124]. Since MMPis are designed to recognize the active site of MMPs, the binding of radiolabeled MMPis thus serves as an activity-based probe for MMP activity *in vivo*. So designing the compound to recognize distinct features of the active site of individual MMP family members gives an excellent selectivity.

 Initial attempts to develop radiolabeled MMPis have shown unsuccessful *in vivo* results. Labeled potent MMPis such as [^11^C]MSMA and [^11^C]CGS25966 showed high levels of nonspecific binding in mouse models of breast cancer [125]. Evaluation of [^11^C]FMAME, a molecule developed by the same group, concluded nonspecific binding [126]. [^18^F]SAV03, another MMPi labeled with ^18^F along with its methyl ester derivative, [^18^F]SAV03M, which is used as a prodrug, showed significantly higher uptake in tumor tissue than other organs in biodistribution studies [127]. The tumor accumulation of radioactivity observed in whole body autoradiography with [^18^F]SAV03M promised the possibility of using this agent for visualizing tumors by PET. The radioiodinated MMPis ^123^I- and [^125^I]^ 125^I-CGS 27023A, ^123^I- and ^125^I-HO-CGS 27023A by Kopka et al. showed good *in vitro* affinities towards MMP2 and MMP9 [128]. They observed rapid blood and plasma clearance of the ^125^I-labeled CGS compounds which supports the concept of utilizing these radiotracers for imaging MMP activity [129]. There have also been reports on labeled tryptophan- and valine-based biphenylsulphonamide MMPis, which showed effective inhibition and selectivity for MMP2 [130, 131]. But *in vivo* results failed the expectations, with poor tumor uptake in nude mice bearing A549 lung tumors.

Recently there was a report on Marimastat, a noncovalent MMPi labeled with ^18^F. In the reported method, shelf-stable arylboronic esters conjugate was used as a captor for aqueous ^18^F fluoride in a novel method. Developed tracer was localized to the tumors [132]. Despite a relatively low signal-to-noise ratio indicated by PET imaging, tumor labeling was specific to target. This report is important mainly because of novel labeling methodology rather than *in vivo* application of labeled MMPis. There are also reports on usage of ^99m^Tc-labeled broad-spectrum MMPi, RP-805, to evaluate correlation of macrophage apoptosis and MMP release *in vivo *[133]. Atherosclerosis was produced in rabbits receiving a high cholesterol diet (HC), who underwent radionuclide imaging. MMPi uptake was best visualized in HC diet animals and reduced significantly after fluvastatin treatment or diet withdrawal. There was a significant correlation between ^99m^Tc-MMPi and ^111^In-Annexin A5 (AA5) uptake; both correlated with pathologically verified MMP-9 activity and macrophage content. It should be noted that several studies using ^18^F-conjugated MMPis for *in vivo* imaging also revealed high uptake of tracer in tissues with known MMP overexpression such as the liver and blood [127, 134]. This is a particular problem for broad-spectrum MMPis and leads to poor target/nontarget contrasts when imaging diseased tissues. With more data becoming available on the expression and activity of specific MMPs in particular pathologies, antibodies, or small molecule inhibitors with narrow specificity can be developed as future molecules [132]. There have been reports of using endogenous inhibitors of MMPs, the tissue-specific inhibitors of matrix-metalloproteinases (TIMPs). A clinical SPECT imaging study on five patients with Kaposi's sarcoma (KS) using N-TIMP-2-DTPA labeled with ^111^In was disappointing in that none of the patients showed significant uptake in KS lesions [135].

### 3.2. uPAR

 Urokinase-type plasminogen activator (uPA) and its cell-surface receptor (uPAR) are central molecules for cell surfaces-associated plasminogen activation [136, 137]. The uPA/uPAR system has an important role in cancer progression and metastasis [138]. uPA is a glycosylated serine protease that catalyzes the conversion of plasminogen to plasmin. Binding of pro-uPA to uPAR (CD87) results in proteolytic activation [136, 139]. Binding of uPA to uPAR serves to focalize uPA activity to facilitate invasion of uPAR expressing cancers by activation of a proteolytic cascade that breaks down extracellular matrix components and allows cancer cell migration into vasculature and lymphatics [136]. Also, the uPA/uPAR system is involved in regulating cell-extracellular matrix interactions by acting as an adhesion receptor for vitronectin and by modulating integrin function [139]. These properties of the uPA/uPAR system attracted researchers to use it as a cancer therapeutic target [137].

Li et al. demonstrated the noninvasive imaging of uPAR expression in a living subject for the first time by using a small linear peptide (D-Cha-F-s-r-Y-L-W-S) (AE105) with high affinity for human uPAR in uPAR xenotransplanted mouse tumor models after labeling with the positron emitter ^64^Cu [140]. *In vivo* PET imaging analysis in high levels of uPAR expressing U87MG glioma cells and uPAR-negative MDA-MB-435 breast cancer cells revealed gradual increase of ^64^Cu-DOTA-AE105 uptake in U87MG tumors with time and uptake that remained close to background in the case of MDA-MB-435 tumors. To confirm the receptor specificity, the author studied the imaging of ^64^Cu-DOTA-AE105mut (AE105mut, D-Cha-F-s-r-Y-L-E-S) in U87MG tumor-bearing mice. The aromatic side chain residue of the tryptophan residue in AE105 is indispensable for the high-binding affinity toward uPAR as reported previously [136, 141]. The glutamate replacement in this residue was accompanied by only minimal uptake in U87 tumor. Although the specificity in targeting uPAR with the ^64^Cu-DOTA-AE105 is proven in this study, it is not clear whether this radiotracer actually would allow a quantitative measurement of receptor expression *in vivo*. Also, the liver uptake of ^64^Cu-DOTA-AE105 is relatively high, which may limit its applicability to measure lesions in the liver and intestines. Therefore this promising study needs to improve the pharmacokinetics of this radiopharmaceutical. The author suggested the possibility of using a soluble pseudosymmetrical dimer of AE105 displaying a higher affinity for uPAR and increased solubility [141]. It has been shown that uPA-uPAR binding is species specific with little cross-reactivity between human and murine proteins [142]. A similar selectivity was observed in the case of AE105 as revealed by binding studies using surface plasmon resonance. Hence PET imaging studies of xenotransplanted human tumors in mice used in this study are most unlikely to bind mouse uPAR expressed by the tumor-associated murine stroma and vasculature cells. There was also a report on usage of a dimeric peptide most closely related to peptide antagonist (D-X-F-s-r-Y-L-W-S-G)2-*β*-A-K (AE120), modified by the addition of DOTA C-terminal to a branching lysine residue [141]. Knör et al. subsequently used it in an *in vivo* model of human ovarian cancer and demonstrated tumor uptake of the ^213^Bi complex of 2.2% (0.4% ID/g at 90 min after injection) [143]. But the specificity of tumor uptake of the ^213^Bi-labeled peptide *in vivo* was not addressed.

 In a subsequent study, Liu et al. synthesized and characterized a small peptide inhibitor of the uPA-uPAR interaction and modified it to contain a C-terminal DOTA chelating moiety for labeling with ^111^In to obtain (NAc-dD-CHA-F-dS-dR-Y-L-W-S-Ala)2-K-K([^111^In]-DOTA) [144]. The *in vivo* biodistribution comparison profile to that of ^125^I-amino terminal fragment (ATF) in mice bearing MDA-MB-231 human breast cancer xenograft showed significantly different data at all time points examined. At 1 and 4 h post injection, blood levels of each radiotracer were distinctly different, with the higher molecular weight ATF fragment demonstrating significantly slower clearance. ^125^I-ATF uptake was higher in all normal tissues at these time points, with the exception of liver and kidney, which demonstrated higher levels of ^111^In-peptide. Increased liver and kidney retention was observed for the ^111^In-labeled branched peptide relative to ^125^I-ATF at 24 h. The tumor uptake value shown by this peptide was similar to Knör et al. [143], although the values are not directly comparable due to differences in the model system, radioisotope, and experimental protocol.

## 4. Targeting Tumor Hypoxia

Hypoxia can occur due to structural abnormalities of microvessels and the limited diffusion distance (<70 mm) of oxygen within the tumor. Increase in tumor aggressiveness and metastatic potential of solid tumors is believed to be highly associated with the presence of hypoxia within the cancer [145]. Tumors often adapt to hypoxic environments by upregulation of the HIF-1, a heterodimer protein composed of oxygen-sensitive HIF-1*α* and constitutively expressed HIF-1*β* subunits. When stabilized by hypoxic conditions, HIF-1 binds and transactivates several genes associated with enhanced glycolysis and angiogenesis. Due to these changes, patients with hypoxic tumors often have a poor prognosis and decreased overall survival rate due to higher degrees of invasiveness and resistance to chemo- and radiation therapy [146–148]. These limitations drive significant importance to the need to detect hypoxia within tumors for cancer management. Currently, the eppendorf needle electrode system has been used for the direct measurement of oxygen level in tumors, which is limited only to easily accessible tumors. There are limitations in direct measurement of hypoxia because they are invasive procedures and may be subject to sampling error; additionally, it is difficult to reach all tissue sites, and thus measurements are only accessible at the time of surgery. Because of the heterogeneous nature of hypoxia in tumors, substantial interest has been paid to the development of noninvasive techniques that permit serial noninvasive imaging of hypoxia, which could extract valuable information on disease that is required in oncology applications.

**Figure 6:**
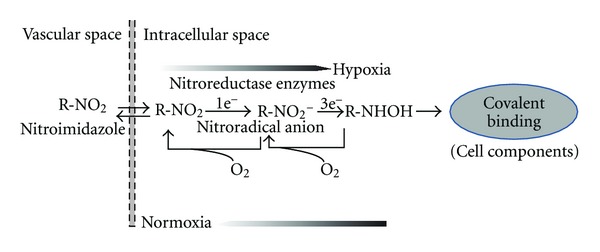
Schematic representation of the proposed mechanism for the binding of nitroimidazole radiotracers in hypoxic environment.

 For the hypoxia detection, the tracer should be specific for hypoxia, its uptake should reflect clinically relevant cellular *p*_O_2__ values (0–10 mmHg) irrespective of the tumor type and grade, and it should easily cross the blood capillary membrane, preferably without using membrane transporter systems, which otherwise might complicate the interpretation of imaging results. Thus, rapid membrane permeation and localization to the viable hypoxic target tissue with high specificity is essential. Although none of the currently available tracers have all the properties of an ideal hypoxia imaging agent, the selection is based largely on tumor type, ease of synthesis, and availability of radioisotope. 2-Nitroimidazole, which is reduced under hypoxic conditions and consequently accumulates in sites of hypoxia, has been an attractive molecule for many years for PET or SPECT imaging purposes after labeling with different radioisotopes [15, 148, 149]. Under hypoxic conditions, the nitroimidazole molecule undergoes an enzymatic single electron reduction, depending on the availability of oxygen, which forms a radical anion (Figure 6). The process is initiated by an enzyme-mediated single electron reduction to form a free radical. The rate of oxidation is dependent on the intracellular concentration of oxygen. In hypoxic tissue, the reduced compound is not able to be oxidized; instead, it is further reduced and binds to intracellular components.

**Figure 7:**
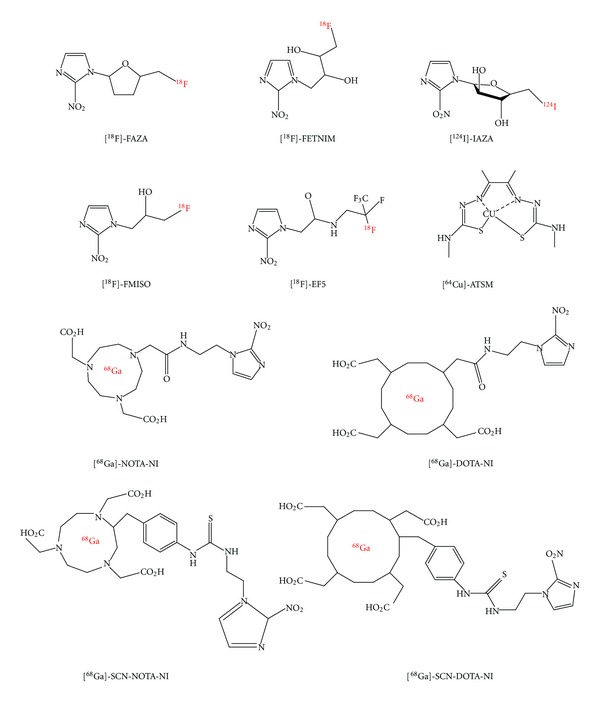
Chemical structures of radiolabeled hypoxia imaging agents. ^18^F-fluoroazomycin arabinoside ([^18^F]FAZA) [13], ^18^F-fluoroerythronitroimidazole ([^18^F]FETNIM) [14], ^123^I-iodoazomycin arabinoside ([^123^I]IAZA) [15], 1-^18^F-fluoro-3-(2-nitro-1*H*-imidazol-1-yl)propan-2-ol ([^18^F]FMISO) [16], 2-^18^F-2-[2-nitro-^1^H-imidazol-1-yl]-N-(2,2,3,3,3-pentafluoropropyl)acetamide ([^18^F]EF5) [17], ^64^Cu-diacetyl-bis (^4^ *N*-methylthiosemicarbazone) (^64^Cu-ATSM) [18], ^68^Ga-NOTA-nitroimidazole (^68^Ga-NOTA-NI) [19], ^68^Ga-DOTA-nitroimidazole (^68^Ga-DOTA-NI) [19], ^68^Ga-NOTA-SCN-nitroimidazole (^68^Ga-NOTA-SCN-NI) [20], and ^68^Ga-DOTA-nitroimidazole (^68^Ga-DOTA-SCN-NI) [20].

Detection of tumor hypoxia with radionuclides was first demonstrated with [^14^C]-misonidazole by autoradiography [150], which was later proposed for noninvasive PET imaging of tumor hypoxia after labeling with ^18^F. Since then, several other tracers which include [^18^F]fluoroerythronitroimidazole (FETNIM), [^18^F]fluoroazomycin arabinoside ([^18^F]FAZA), ^123^I-iodoazomycin arabinoside ([^123^I]IAZA), ^64/62/60^Cu-diacetyl-bis(^4^ *N*-methylthiosemicarbazone) (^64^Cu-ATSM), ^99m^Tc-butylene amineoxime (^99m^Tc-HL91), and 2-(2-nitro-1*H*-imidazol-1-yl)-N-(2,2,3,3,3-[^18^F]pentafluoropropyl) acetamide ([^18^F]EF5) have been evaluated for this purpose (Figure 7).

^18^F-misonidazole ([^18^F]FMISO) has been used for PET imaging quantification of hypoxia in a variety of tumors, including head and neck cancer, non-small-cell lung cancer, breast cancer, and brain tumors [16]. These studies have shown tumor-to-background ratio of at least 0.88–5.85 after 2 to 3 h after injection. One major limitation of this tracer is slow clearance from nonhypoxic tissues. [^18^F]FAZA was developed to undergo more rapid clearance from blood and nontarget tissues than [^18^F]FMISO [13]. Because of these improved imaging properties, [^18^F]FAZA is recommended for further preclinical and clinical study for imaging tumor hypoxia in various tumors including lymphoma and gliomas [151]. Compared with [^18^F]FMISO, [^18^F]FAZA displays a higher tumor-to-background ratio (1.2 to 15.5). Also, the significantly lower tumor-to-blood ratio resulting from [^18^F]FAZA is related to either renal or hepatobiliary excretion, leading to a lower radiation burden and to a favorable imaging result compared with [^18^F]FMISO [13]. In patients with glioblastoma multiforme, [^18^F]FAZA yielded high tumor-to-background ratios due to selective and presumably hypoxia-specific uptake in tumors reflecting blood-brain-barrier disruption. It has been reported that [^18^F]FAZA PET can be used to define the target volume for dose increase in radiation treatment planning [152]. Even though [^18^F]FAZA shows significant promise for hypoxia imaging, there is no direct comparison with [^18^F]FMISO in patients. [^18^F]EF5 is one of the most liphophilic 2-nitroimidazole derivatives developed for hypoxia imaging. The lipophilicity of the compound was enhanced by introducing five fluorine atoms to the nitroimidazole side chain, which could also increase its biological half-life. This tracer has unique advantages, including its use as a fluorescence immunohistochemistry marker for hypoxia in nonradioactive form. Also, its high *in vivo* stability is an additional advantage. In the case of both [^18^F]FAZA and [^18^F]EF5, the time required after injection to obtain good images is 2 to 3 h. The first human study of [^18^F]EF5 showed hypoxia-specific tumor binding in head and neck cancer [153].

^64^Cu-ATSM is a nonnitroimidazole compound developed for hypoxia imaging [18]. The use of copper-labeled radiopharmaceuticals for PET is attractive because of the increasing availability of four positron-emitting radionuclides of copper, such as ^60^Cu (*t*_1/2_ = 0.40 h, *β* ^+^ = 93%, EC = 7%), ^61^Cu (*t*_1/2_ = 3.32 h, *β* ^+^ = 62%, EC = 38%), ^62^Cu (*t*_1/2_ = 0.16 h, *β* ^+^ = 98%, EC = 2%), and ^64^Cu (*t*_1/2_ =  12.7 h, *β* ^+^ = 17.4%, EC = 43%) [154]. ^62^Cu can be produced by generator (^62^Zn/^62^Cu generator) system [155, 156], whereas ^60^Cu, ^61^Cu, and ^64^Cu are produced by cyclotron [157, 158] using reliable and reproducible targets. In a hypoxic environment, Cu(II)-ATSM can be trapped intracellularly after the one-electron reduction, which resulted in Cu(I)-ATSM. It shows high tumor-to-background ratios (1.0 to 10.4) in less than 1 h after injection with rapid delineation of tumor hypoxia. It has been shown to be selective for hypoxic cancers and ischemic myocardial tissue [159]. A comparative biodistribution study in mice bearing EMT6 tumors with ^64^Cu-ATSM, ^64^Cu-pyruvaldehyde-bis(*N* ^4^-methylthiosemicarbazone) (PTSM), and ^18^F-MISO showed optimal tumor uptake of both agents after 10 min after injection, suggesting a rapid trapping mechanism for these agents in solid tumors [160]. *Ex vivo *autoradiography of tumor slices after coinjection of ^64^Cu-ATSM and ^64^Cu-PTSM into the same animal was also studied. The results showed uniform spreading of ^64^Cu-PTSM throughout the EMT6 tumor compared to heterogeneous uptake of ^64^Cu-ATSM, suggesting that ^64^Cu-ATSM has better selectivity for hypoxia imaging. Clinical studies have shown that ^60^Cu-ATSM can predict tumor response to therapy in different types of cancers [161]. A direct comparison of ^60^Cu-ATSM and ^64^Cu-ATSM scans in cervical cancer patients showed similar patterns and magnitudes of uptake, but ^64^Cu-ATSM produced better-quality images than ^60^Cu-ATSM due to low noise [162].

**Figure 8:**
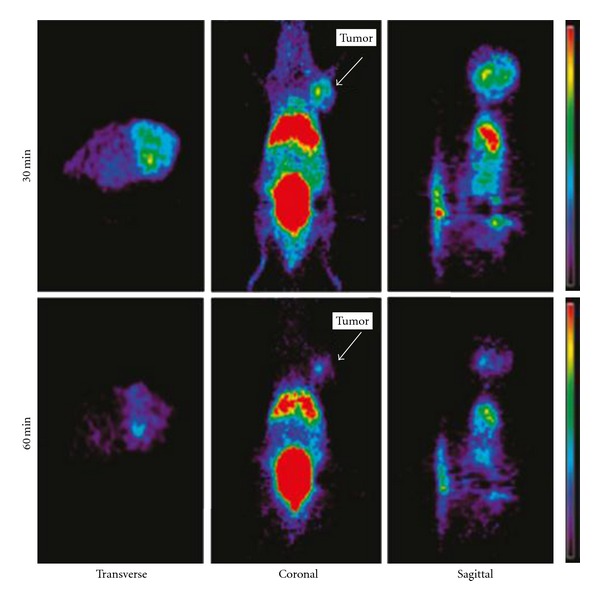
^68^Ga-NOTA-NI microPET images of mice bearing a CT-26 tumors after intravenous administration of ^68^Ga-NOTA-NI (13.3 MBq/0.1 mL) (30 and 60 min after injection). Arrows indicate tumor positions [19].

However, the production of these radiotracers is limited to cyclotron systems, which are expensive as well as difficult to handle. Other than the cyclotron produced radioisotopes, an alternative method to label biomolecules is the use of ^68^Ga, which can be obtained from a commercially available radionuclide generator system [7, 163–168]. ^68^Ga is an economical alternative to the cyclotron produced radionuclides. Compounds characterized by high hydrophilicity were thought to be better for imaging hypoxia because of rapid blood clearance and high target-to-nontarget ratio. Recently, ^68^Ga-labeled nitroimidazole (NI) analogues were developed [19, 20] for PET hypoxia imaging (Figure 8). These derivatives showed elevated uptake in hypoxic conditions compared to normoxic conditions in both *in vitro* and *in vivo* studies.

## 5. Targeting Tumor Growth and Recruitment/Homing

### 5.1. Receptor Tyrosine Kinase

**Figure 9:**
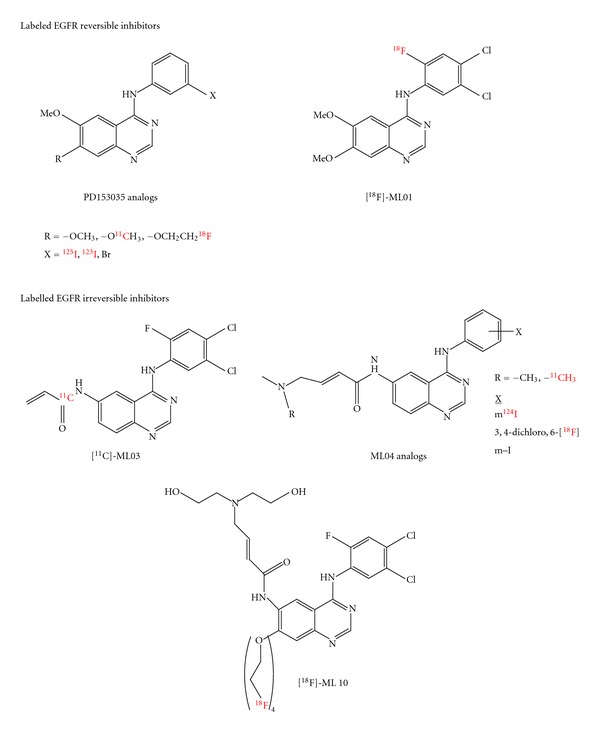
Chemical structures of EGFR inhibitors used for radiolabeling. Labeled EGFR reversible inhibitors (PD153035 analogs and [^18^F]ML01) [21, 22] and labeled EGFR irreversible inhibitors (ML04 analogs, [^11^C]ML03, and [^18^F]ML10) [23–25].

 Because of the importance of the epidermal growth factor receptor (EGFR) signaling pathway in malignant progression of various types of tumors [169], there has been growing interest in the use of EGFR-TK inhibitors as probes for molecular imaging of EGFR overexpressing tumors *via* PET/SPECT [170–172]. Such noninvasive and repetitive monitoring of the activity of EGFR at the kinase level could provide a direct measure of EGFR occupancy and inhibition by EGFR-targeting drugs. These observations led several research groups to develop novel radiolabeled EGFR-kinase-specific agents for SPECT and PET imaging. Most of these agents are derivatives of quinazolines that reversibly or irreversibly bind to the EGFR ATP-binding pocket (Figure 9). Noted agents include a reversible 4-[(3,4-dichloro-6-[^18^F]fluorophenyl)amino]-6,7-dimethoxyquinazoline (ML01), which was tested in an EGFR overexpressing subcutaneous tumor [21]. In another study, the biodistribution of [^11^C]-labeled 4-(3-bromoanilino)-6,7-dimethoxyquinazoline ([O-^11^C-methyl]PD153035) has been evaluated in rat neuroblastoma xenografts [22]. Both these studies concluded that, because of the reversibility of binding to EGFR, these radiotracers were rapidly washed out of tumor tissue without generating an adequate target-to-background ratio. To improve the radiotracer retention at the EGFR kinase site, a N-4-[(4,5-dichloro-2-fluorophenyl)amino]quinazolin-6-yl-[^11^C]acrylamide ([^11^C]ML03) was developed [23]. Despite some improvement in tumor targeting, the very short half-life of ^11^C (20 min) limits opportunity for imaging. With these studies, the authors concluded that effective EGFR imaging agents based on inhibitors that irreversibly bind only to the active form of EGFR kinase labeled with longer-lived radionuclides are required to allow for sufficient washout of nonspecific radioactivity and development of higher signal-to-background ratios. Due to limitations of reversible inhibitors as imaging agents, researchers shifted their focus towards the design and development of novel irreversible inhibitors as imaging candidates [23, 170].

Inhibitors like [4-(3-iodoanilino)-quinazolin-6-yl]-amide-(3-morpholin-4-yl-propyl)-amide (ML04) [22] and 4-dimethylamino-but-2-enoic acid [4-(phenylamino)-quinazoline-6-yl]-amide [24] reported better pharmacologic profiles than previously studied irreversible inhibitors [23]. The biodistribution of ^18^F-ML04 was evaluated in nude mice bearing human glioma xenografts, U87MG-wtEGFR [24]. After 3 h after injection, tracer activity in the tumor was 2–7-fold higher than other organs. Although the *in vivo *pharmacology and stability of ML04 was much better than other reported irreversible radiolabeled EGFR kinase inhibitors, PET images in tumor-bearing animal models remained suboptimal. Inefficient formation of covalent adducts with EGFR kinase domain could be the reason for these observations. The interactions of [4-(3-[^124^I]iodoanilino)-quinazolin-6-yl]-amide-(3-morpholin-4-yl-propyl)-amide, the morpholino-^124^IPQA to the ATP-binding site of activated EGFR and covalent adduct were also analyzed. Studies confirmed that the extension of a side chain from the 6-position of [4-(3-halogeno-anilino)-quinazoline] pharmacophore does not significantly impact the EGFR kinase inhibitory activity of this class of compounds. There are also promising studies on PET imaging using the morpholino-[^124^I]-IPQA in rats bearing dual tumor xenografts [171]. However, despite the lower lipophilicity of morpholino-[^124^I]-IPQA, it still exhibited a significant hepatobiliary clearance. Towards the further optimization of aminophenyl-quinazoline-based agents, Dissoki et al. had reported several analogues of quinazoline with short polyethylene glycol (PEG) side chains (Figure 9) [25]. These derivatives were labeled with ^18^F at the terminal positions of the PEG chain. Labeled compounds showed a decreased potency of inhibition along with increased length of PEG side chain. Also, studies revealed the better possibility of PEGylated ML04 derivatives for molecular imaging of EGFR-positive tumors than ML04.

 Levashova et al. reported dEGF (a Cys-tagged dimeric EGF)-based SPECT tracer prepared by direct radiolabeling of Cys-tag with ^99m^Tc, while the corresponding PET tracers were prepared by conjugating a PEGylated DOTA chelator to Cys-tag, followed by radiolabeling with ^64^Cu [173]. PET tracers were evaluated in a luciferase-positive MDA-MB-231 breast tumor and observed clear accumulation in large (10–15 mm) size tumor at 3 h after injection. Also, SPECT tracers were tested in breast carcinoma and spontaneous mouse lung carcinoma models [174, 175]. In both models, the tracers showed significant accumulation in the tumors. More recently there has been a report on radiohalogenated 4-anilinoquinazoline-based EGFR-TK inhibitors as potential cancer imaging agents [176]. *In vitro* results showed micromolar inhibition of EGFR autophosphorylation and EGFR expressing cell proliferation. But poor ^18^F labeling yield of the most effective compound in this series hampered further biological evaluation.

### 5.2. Somatostatin Receptors

 Somatostatin is a regulatory peptide and its action is mediated by membrane-bound receptors (SSTRs), G-protein-coupled receptors, that are highly expressed in many different types of human tumors, notably neuroendocrine tumors (NET) [177], which in clinical practice are usually carcinomas and pheochromocytomas. SSTRs are also expressed, to variable extents, in renal cell carcinoma, small cell lung cancer, breast cancer, prostate cancer, and in malignant lymphoma [163]. There are five SSTR subtypes, but subtype 2 (SSTR2), subtype 5 (SSTR5), and to a lesser extent, subtype 3 (SSTR3) have higher affinities than SSTR1 and 4, and thus, commercially available synthetic somatostatin analogues target these three high affinity receptors [178]. These analogues are required because somatostatin is rapidly degraded by enzymes *in vivo*, as reflected by its short biological half-life, and thus, agents with high affinity for SSTR have been developed which are resistant to enzyme degradation. Somatostatin analogues, such as DOTA-TOC, show better images than ^111^In-DTPA-octreotide, the most commonly used somatostatin analogue [179]. The phenylalanine residue at position 3 was replaced by tyrosine in DOTA-TOC, which makes the compound more hydrophilic, increases affinity for SSTR2, and increases uptake by SSTR2-positive tumors [180]. Other peptides have also been linked to DOTA, such as DOTA-octreotate, which has high affinity for SSTR2 [178], and DOTA-lanreotide, which has high affinity for SSTR5. DOTA-NOC is the newest addition to these compounds and has high affinity for SSTR2, SSTR3, and SSTR5. Furthermore, these DOTA-peptide products show high radiochemical purity, rapid renal clearance, and high accumulation in tumors, and overall represent remarkable advances over standard peptides [181]. Antunes et al. demonstrated that gallium ^67^Ga- and ^68^Ga-DOTA-octapeptides have distinctly better preclinical pharmacological performance than ^111^In-labeled peptides, especially on SSTR2-expressing cells and in animal models [163]. In particular, ^68^Ga-DFO-octreotide injected into rats bearing SSTR-positive pancreatic tumors demonstrated selective binding to tumor sites with a tumor to background ratio of 5 [182]. Subsequently, several DOTA-SST analogues were evaluated *in vivo*, and ^68^Ga-DOTA-TOC and ^68^Ga-DOTA-NOC were found to be the most promising [183–186]. Radiotherapy with ^111^In-labeled somatostatin analogues was attempted, but only occasional objective tumor responses were observed [187, 188]. Also, using the high energy beta emitter ^90^Y, labeled with DOTA-[Tyr3]-octreotide, partial tumor remission was achieved [189, 190]. The improved therapeutic efficacy was achieved by ^177^Lu-labeled [DOTA,Tyr3,Thr8]-octreotide, which has higher affinity for sstr2 than [DOTA,Tyr3]-octreotide [191, 192].

^68^Ga-based PET tracers (^68^Ga-DOTATOC, ^68^Ga-DOTATATE, and ^68^Ga-DOTANOC) are widely used, in which ^68^Ga-DOTANOC has affinity for sst2, sst3, and sst5, resulting in a better diagnostic sensitivity than ^68^Ga-DOTATATE, which has the highest sst2 affinity [193]. Studies comparing the ^68^Ga-based peptides with ^111^In-labeled octreotide showed the PET images with distinctly higher sensitivity [194]. Most recently, several phase I and II clinical studies on targeted radionuclide therapy using different combinations have been reported [195, 196]. The variability in the therapeutic injected dose, number of treatment cycles, and recruited patients population makes the comparison of these data very complex. The ^90^Y-labeled DOTA-lanreotide was investigated in 39 patients, but no objective response was noticed [197]. Clinical data on ^90^Y-DOTATATE show an objective response rate of 37% [198]. Other clinical studies using ^90^Y-DOTATOC in 60 patients resulted in 23% objective response rate [199]. They also report on ^177^Lu-DOTATATE therapy in 35 patients initially and in follow-up studies with 310 patients, which shows around 30% objective response rate [200, 201]. Recently, a very exciting combination therapy approach was studied using ^90^Y-DOTATATE in tandem with ^90^Y/^177^Lu-DOTATATE. The therapy with tandem radioisotopes provided longer overall survival than with a single radioisotope and the safety of both methods was comparable [202]. Efforts are continuing to improve the therapeutic efficacy of somatostatin derivatives labeled with therapeutic radionuclides.

### 5.3. Chemokine Receptor

 The chemokine receptors, which belong to a family of seven transmembrane domain G-protein-coupled receptors, consist of 18 members [203]. Numerous studies have reported that CXCR4 among all chemokine receptors plays a critical role in tumor invasion and metastasis by interacting with its ligand stromal cell-derived factor-1 (SDF-1 or CXCL12), which is highly expressed in common destination organ sites of metastasis [204, 205]. The CXCR4/SDF-1 interaction and the resulting cell signaling cascade have emerged as highly relevant targets since they play pleiotropic roles in metastatic progression, especially homing. CXCR4 is overexpressed in more than 20 different human cancers [206, 207], and therefore has been proposed as a prognostic factor and therapeutic target [208, 209]. In initial attempts to image CXCR4 expression in cancer models, researchers evaluated ^111^In-labeled peptides and ^125^I-labeled monoclonal antibodies with SPECT/CT [210, 211]. Hanaoka et al. designed a 14-residue peptide inhibitor, Ac-TZ14011 and evaluated it in nude mice bearing the CXCR4-expressing pancreatic carcinoma AsPC-1, after labeling with ^111^In [210]. Results showed greater accumulation of radioactivity in the tumor than in the blood or muscle. The authors found that conjugation with diethylenetriaminepentaacetic acid (DTPA) and labeling with ^111^In reduced the specificity of the analog to CXCR4 by 6-fold. Another group of researchers opted to label the endogenous ligand for CXCR4, SDF-1*α* with ^99m^Tc, for quantitation of CXCR4 expression in myocardial infarction. These findings revealed 5-fold higher levels of CXCR4 in infarcted myocardium subjected to ischemic injury than noninfarcted areas at 24 h after injury. ^99m^Tc-labled SDF-1*α* showed high affinity and specificity for its endogenous chemokine receptor [212]. CXCR4 also proved to have an important role in brain tumor development, growth, and metastasis. To evaluate CXCR4 expression in U87MG xenografts, researchers generated a radiolabeled version of a mouse antihuman CXCR4 antibody (12G5) and imaging was conducted using SPECT/CT [211]. In imaging and biodistribution studies, highest uptake was seen in the spleen, followed by the tumor. The tumor-derived cells showed a 2- to 7-fold increase in surface CXCR4 expression in flow cytometry results, which indicates the significant retention of radioactivity observed in larger tumors due to ^125^I-12G5 binding to CXCR4 and not by enhanced permeability and retention. These results demonstrate the feasibility of imaging tumor microenvironment induced CXCR4 expression, to provide an indirect readout on the heterogeneity of tumors.

**Figure 10:**
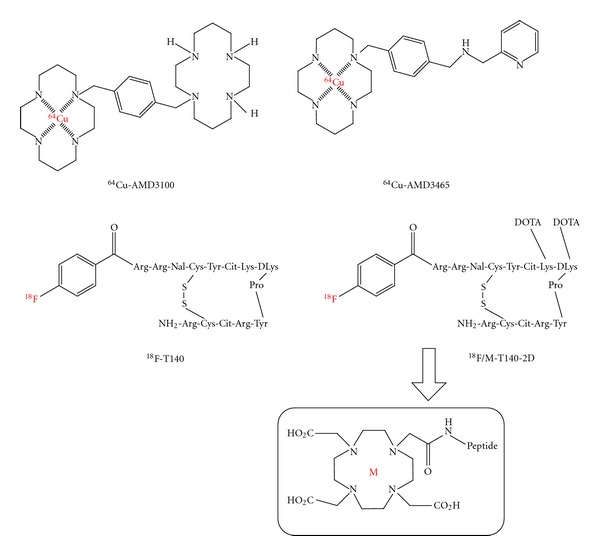
Structures of radiotracers developed for imaging CXCR4 expression in tumors. ^64^Cu-labeled small molecule CXCR4 inhibitors (^64^Cu-AMD3100 and ^64^Cu-AMD3465) [26, 27] and ^18^F-labeled peptide inhibitors (^18^F-T140 and ^18^F/M-T140-2D) [28, 29].

Recently, Nimmagadda et al. imaged CXCR4 expression in two human breast cancer cell lines with ^64^Cu-labeled CXCR4 inhibitor AMD3100 (Figure 10) [26]. However, the author attributed high liver and background tissue uptake to basal levels of CXCR4 expression and moderate plasma protein binding (58%) observed with AMD3100. Additionally, the bicyclam AMD3100 has a relatively low affinity (~651 ± 37 nM) and a structurally restricted scaffold. The same group pursued their research with monocyclam analog N-[1,4,8,11-tetraazacyclotetradecanyl-1,4-phenylenebis(methylene)]-2-(aminomethyl)pyridine (AMD3465) to image CXCR4 expression (Figure 10) [27]. Compared with AMD3100, AMD3465 has higher affinity, reduced size, and charge [213]. AMD3465 was shown to be 10-fold more effective as a CXCR4 antagonist than the bicyclam AMD3100 [214]. The specificity, target selectivity, and tumor-to-muscle ratios observed suggest that ^64^Cu-AMD3465 is a better agent for *in vivo* PET, compared with other known agents. Imaging studies clearly demonstrated selective accumulation of radioactivity in the CXCR4-positive U87MG tumors. The tumor-to-muscle and tumor-to-blood ratios for ^64^Cu-AMD3465 at 90 min after injection were 7- to 8-fold higher than those of ^64^Cu-AMD3100. Even though ^64^Cu-AMD3465 has improved affinity and kinetics, considerable uptake in the liver and kidneys remains a concern.

 There are also recent reports on labeling of CXCR4 peptide antagonist T140 (Figure 10) with ^18^F and its evaluation *in vivo *as a CXCR4 imaging agent [28]. Results show that, when injected in high specific activity (with no addition of unlabeled mass of peptide), ^18^F-T140 bound to mouse RBCs and gave high background signal. But binding to the RBCs was blocked by coinjection of the tracer, also resulting in elevated accumulation in CXCR4 positive tumor. Apart from these drawbacks, radiosynthesis of ^18^F-T140 requires several steps, a long reaction time and results in low yield, limiting its potential for clinical use. The same group of researchers simplified the synthesis of T140-based tracer by conjugation of 1,4,7,10-tetraazacyclododecane-1,4,7,10-tetraacetic acid mono (*N*-hydroxysuccinimide ester) (DOTA-NHS) and labeled with ^64^Cu to generate ^64^Cu-T140-2D derivative (Figure 10) [29]. Even though radiosynthesis is simplified, ^64^Cu-T140-2D showed similar behavior as ^18^F-T140 in terms of binding to mouse RBCs and human RBCs. Also, significantly higher uptake in the kidney and liver over time compared to ^18^F-T140 remains a concern. The same studies with ^68^Ga labeling ended with similar results. All these findings reveal the need of additional considerations to develop successful CXCR4 imaging agents. Most recently Gourni et al. evaluated the ^68^Ga-labeled high-affinity cyclic CXCR4 ligand, ^68^Ga-CPCR4-2 (cyclo(DTyr1-[NMe]-D-Orn2-[4-(aminomethyl) benzoic acid,^68^Ga-DOTA]-Arg3-2-Nal4-Gly5)) [215]. Labeled ligand showed high *in vivo* stability with high and specific tumor accumulation, which was reduced by approximately 80% in competition studies with AMD3100.

## 6. Summary

Molecular imaging will play a key role in shaping 21st century cancer management. Cooperative efforts are needed from biologists, chemists, engineers, medical physicists, and mathematicians in identifying, synthesizing, and characterizing excellent imaging probes and in developing high sensitivity imaging instruments. The probes developed for tumor angiogenesis imaging can also have broad applications for other angiogenesis-related diseases, such as myocardial infarction, stroke, atherosclerosis, chronic inflammation, and others. Even though there is still room for improvement, the diagnostic and radiotherapeutic targeting of neuroendocrine tumors with peptide-based nuclear probes has been proven very useful. The development of new radiopeptides with improved pharmacokinetics could help to explore the full potential of targeted radiation therapy. The investigation of integrin *α*_v_ *β*_3_ targeted delivery of radiopharmaceuticals is relatively rare and further research effort is still needed to develop novel integrin-targeted radiotherapy with better tumor targeting efficacy and desirable pharmacokinetics. There is yet to be a clear winner in the approaches used to image *in vivo* MMP activity. There are several technological advances that are likely to make important contributions to the development of MMP-based imaging agents. Although the search for an ideal hypoxia imaging agent is likely to continue, current PET hypoxia imaging methods already show promise for hypoxia-directed treatments. Even though targeting the tyrosine kinase domain of the EGFR for therapy and noninvasive molecular imaging *in vivo* has been in the focus for more than a decade, obtaining a desirable probe remains a challenge. Despite all the challenges and drawbacks, tumor stroma remained as the most desirable target for molecular imaging and therapy for cancer management, as new targets like CXCR4 and uPA/uPAR systems are getting additional interest.
